# Factors influencing the diagnostic and prognostic values of circulating tumor cells in breast cancer: a meta-analysis of 8,935 patients

**DOI:** 10.3389/fonc.2023.1272788

**Published:** 2023-11-27

**Authors:** Hongfang Zhao, Luxuan Wang, Chuan Fang, Chunhui Li, Lijian Zhang

**Affiliations:** ^1^ Clinical Medicine College, Hebei University, Baoding, China; ^2^ Department of Neurosurgery, Affiliated Hospital of Hebei University, Baoding, China; ^3^ Department of Neurological Function Examination, Affiliated Hospital of Hebei University, Baoding, China; ^4^ Postdoctoral Research Station of Neurosurgery, Affiliated Hospital of Hebei University, Hebei University, Baoding, China; ^5^ Key Laboratory of Precise Diagnosis and Treatment of Glioma in Hebei Province, Affiliated Hospital of Hebei University, Hebei University, Baoding, China

**Keywords:** heterogeneity, circulating tumor cell, breast cancer, diagnosis, prognostic value

## Abstract

**Background:**

Circulating tumor cells (CTCs) could serve as a predictive biomarker in breast cancer (BC). Due to its high heterogeneity, the diagnostic and prognostic values of CTC are challenging.

**Methods:**

We searched published studies from the databases of PubMed, Cochrane Library, Embase, and MEDLINE. The detection capability and hazard ratios (HRs) of CTCs were extracted as the clinical diagnosis and prognosis evaluation. Subgroup analyses were divided according to the detection methods, continents, treatment periods, therapeutic plans, and cancer stages.

**Results:**

In this study, 35 publications had been retrieved with 8,935 patients enrolled. The diagnostic efficacy of CTC detection has 74% sensitivity and 98% specificity. The positive CTC detection (CTC**^+^**) would predict worse OS and PFS/DFS in both mid-therapy and post-therapy (HR_OS_, 3.09; 95% CI, 2.17–4.39; HR_PFS/DFS_, 2.06; 95% CI, 1.72–2.47). Moreover, CTC**^+^** indicated poor survival irrespective of the treatment phases and sampling times (HR_OS_, 2.43; 95% CI, 1.85–3.19; HR_PFS/DFS_, 1.82; 95% CI, 1.66–1.99). The CTC**^+^** was associated with poor survival regardless of the continents of patients (HR_OS_ = 2.43; 95% CI, 1.85–3.19).

**Conclusion:**

Our study suggested that CTC**^+^** was associated with a worse OS and PFS/DFS in the Asian population. The detection method, the threshold level of CTC**^+^**, therapeutic approaches, and sampling times would not affect its diagnostic and prognostic values.

## Background

Breast cancer (BC) is the most common cancer and will be the primary leading cause of cancer-related mortality for women in the future (1, 2). Imaging and clinicopathological information are the traditional methods for diagnosis and prognosis assessment (3). However, those evaluations could not reflect the BC condition in real time. Thus, it is difficult to assign optimal treatments (4, 5). Therefore, there is considerable interest in developing more accurate and convenient methods for diagnosis and prognosis assessment.

Liquid biopsy has been considered as a non-invasive approach and utilized comprehensively in cancer research (6). The common analytes of liquid biopsy include circulating tumor cells (CTCs), circulating tumor DNA (ctDNA), and extracellular vesicles (7). CTCs are tumor cells which are shed from the primary cancers or secondary tumors. It could enter into the circulation system and cause secondary cancer formations consequently (8). Previous evidence showed that CTCs could represent tumor progression (9–11). Furthermore, the positive detection of CTCs (CTC**^+^**) in the circulation system could evaluate the survival of the patients (12). There has been an increasing number of literature emphasizing the potential of CTCs having an important role in diagnosis, prognosis, and therapeutic effect assessment in clinical settings (13–16).

Several meta-analyses have explored the relationships between CTC**^+^** and cancer outcomes. In their study involving 2,957 patients from 27 cohorts, Jin and his colleagues revealed that CTCs indicated poor prognoses universally in lung cancers (14). Current studies also showed similar outcomes in hepatocellular carcinoma and pancreatic cancer (15, 16). Although the prognostic and predictive values of CTCs have been verified in many studies, some results were controversial (17). Further investigations are required to identify the factors that influence the diagnostic and prognostic value of CTC**^+^**. Additionally, there is a lack of sufficient research on the diagnostic and prognostic values of CTCs in BC, particularly in relation to the detection methods, therapeutic approaches, and cancer stages (18). Thus, the aims of our study are to investigate the factors that influence CTC**^+^** and to analyze their associations with overall survival (OS), progression-free survival (PFS), and disease-free survival (DFS) of BC patients.

## Methods

This study was prospectively registered in PROSPERO on 08 December 2022 (CRD42022379387) (19) and was performed based on the PRISMA reporting guidelines (**Supplementary Tables 1**, **2**).

### Retrieval strategy and eligibility criteria

The systematic review of the English language articles was conducted based on the PubMed, Cochrane Library, Embase, and MEDLINE databases from 1 January 1970 to 27 April 2023. The detailed search strategies are exhibited in **Supplementary Table 3**. The inclusion criteria were as follows: 1) the clinical sample sources were the peripheral blood samples; 2) the studies provide data of true-positive and false-positive rates for diagnosis detection (3); OS or PFS/DFS was reported as HR of univariate Cox analysis and the 95% confidence interval (CI) was considered valid data; and 4) the patients had BC, whether it had metastasized or not. Both prospective and retrospective observational cohort studies were eligible for inclusion.

### Data extraction

A standard table was constructed for information extraction. Two authors (HF and LX) conducted an independent literature review and recorded their findings. In order to control for selection bias, the authors compared their extracted data at the end of the revision process and resolved any disparities. Duplicate items were removed. If they could not solve the differences, a senior researcher (LJ) performed the data extraction again.

### Main outcomes and study quality assessment

The purpose of this study was to evaluate the diagnostic efficiency of CTC detection and the prognostic value of CTCs. Firstly, we evaluated the quality of all the diagnostic test studies according to the Quality Assessment of Diagnosis Accuracy Studies-2. Secondly, the Newcastle-Ottawa Scale was used to assess the quality of the studies included in the prognosis analysis (20). This scale awarded points based on patient selection (maximum of 4 points), outcome assessment (maximum of 3 points), and comparability of the cohort (maximum of 2 points), with a maximum total of 9 points. The risk of bias was conducted using the Risk of Bias in Non-Randomized Studies of Intervention (Cochrane Bias Methods Group) (21). Publication bias was verified by funnel plot.

### Statistical analysis

The statistical analysis was performed using Stata (Version 12.0). Sensitivity, specificity, and area under the curve were regarded as the gauge of diagnostic analysis. Meanwhile, outcome data were reported as HRs in the prognosis analysis. An HR that exceeds 1 indicated a worse outcome. A higher HR value indicated a poorer prognosis. We estimated study heterogeneity using *I* ^2^ statistics, where greater than 50% was considered significant heterogeneity (*I* ^2^ > 50%). It was preferred to use a fixed-effects model in the absence of significant heterogeneity and a random-effects model in the presence of significant heterogeneity. The *P*-values reported were two-sided, and statistical significance was set at *P <*0.05.

## Results

### Study characteristics for diagnostic and prognostic value analyses

**Figure 1:**
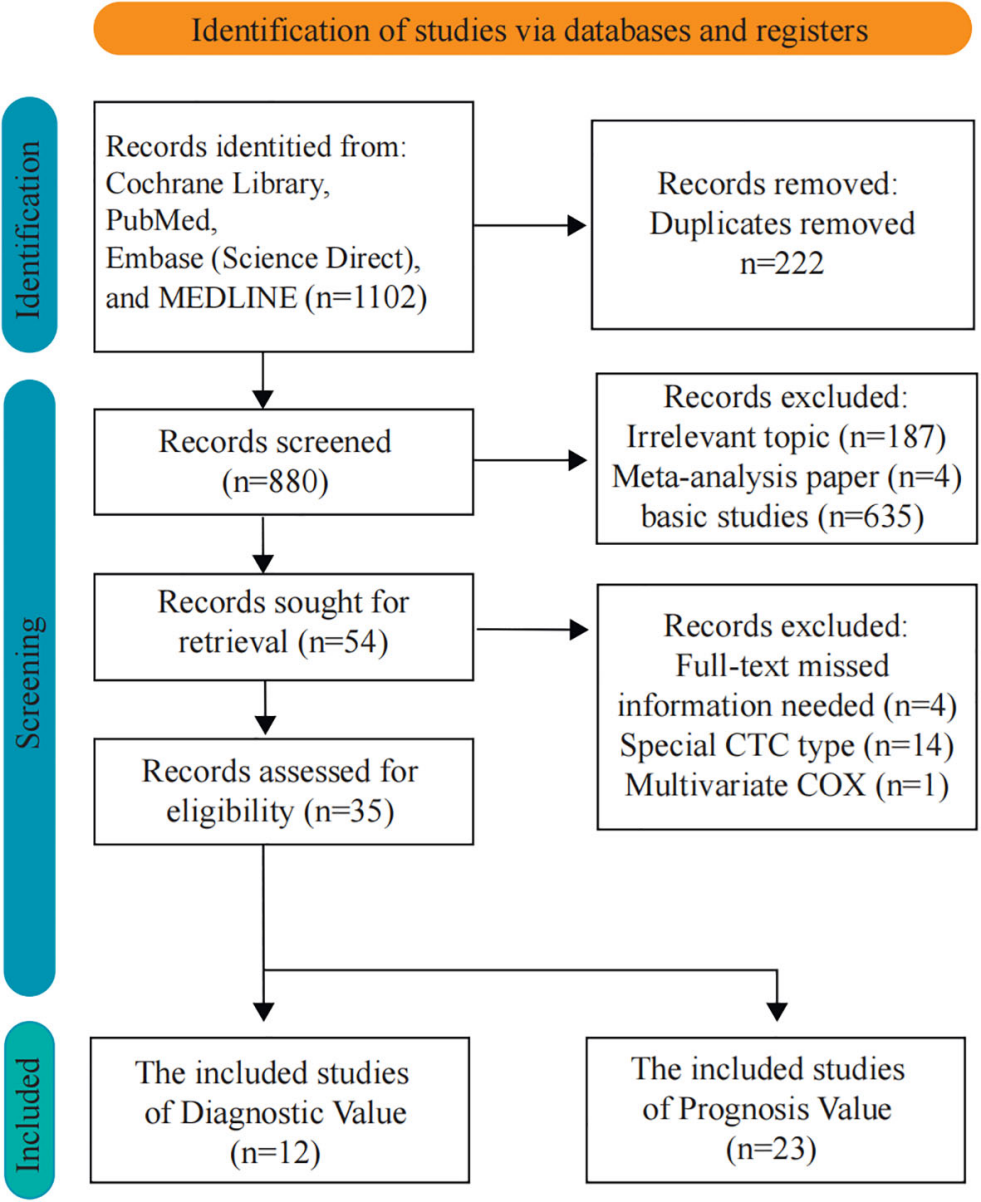
Flow diagram of the study selection for the present meta-analysis.

In this study, the articles were selected according to the diagnostic and prognostic roles of CTCs. There were 1,102 articles obtained from four databases for diagnostic analysis. In order to obtain a comprehensive selection, the full texts of the initially included articles were read entirely. Eight hundred twenty-six studies were unsuitable for inclusion because of duplicate publication. Furthermore, 19 articles were excluded due to missing information, special CTC types, and multivariate Cox which might influence the entire result. Thirty-five articles (8,935 patients) were included after the selection procedure (**Figure 1**). The main features of the eligible studies are summarized in **Tables 1**, **2** (12, 22–55). The QUADAS-2 revised tool and the Newcastle-Ottawa scale were utilized to assess the biases and qualities in the meta-analysis procedure (**Supplementary Tables 3**-**6**).

**Table 1:** Articles included in the diagnostic meta-analysis.

Study	First author of the study (ref.), year	Country	Cancer stage	No. of patients	Detection	TP	FP	FN	TN
1	Riethdorf et al., 2007 (22)	Germany	MBC	237	CellSearch System	53	8	29	137
2	Sawada et al., 2016 (23)	Japan	MBC	42	Fluidic cell microarray chip system	17	9	5	11
3	Sheng et al., 2017 (24)	China	N/A	55	Immunostaining-fluorescence *in-situ* hybridization	41	0	4	10
4	Li et al., 2017 (25)	China	MBC	190	CellCollector	95	0	32	63
5	Jin et al., 2020 (26)	China	ALL	157	CytoSorter system	109	1	19	28
6	Li et al., 2018 (27)	China	ALL	119	NE-FISH platform	85	7	14	13
7	Li et al., 2013 (28)	China	ALL	103	IMPs + ICC	42	0	36	25
8	Weissenstein et al., 2012 (29)	Switzerland	ALL	69	Combination of cytokeratin and EpCAM antibodies	39	0	20	10
9	Zhang et al., 2021 (30)	China	ALL	179	A label-free microfluidic chip	95	9	34	41
10	Kim et al., 2011 (31)	Japan	ALL	77	Telomerase-specific replication-selective adenovirus	21	0	29	80
11	Chen et al., 2010 (32)	China	ALL	100	A three-marker (CK19, hMAM, and CEA) RT-PCR assay	47	1	33	19
12	Zhao et al., 2013 (33)	China	MBC	158	A three-marker (CK19, hMAM, and CEA) RT-PCR assay	86	0	12	60

ICC, immunocytochemistry; IMPs, immunomagnetic nanoparticles; NE-FISH, negative enrichment-fluorescence in-situ hybridization; RT-PCR, reverse transcription-polymerase chain reaction; EpCAM, epithelial cell adhesion molecule; CK 19, cytokeratin 19; hMAM, human mammaglobin; CEA, carcinoembryonic antigen-positive.

Table 2Characteristics of the studies included in the meta-analysis.

**Table:** 

Study ID	First author of the study, year	Country	Continent	No. of patients	Age	Cancer stage	Sampling time	Detection system^a^	CTC^+^ definition^b^
1	Radovich M, 2020 (34)	Indianapolis	America	123	49.6	Early	Mid-therapy	CellSearch System	1 per 7.5 ml
2	Massimo Cristofanilli, 2004 (35)	America	America	177	58	Advanced	Post-therapy	CellSearch System	5 per 7.5 ml
3	François-Clément Bidard, 2021 (36)	France	Europe	377	64	Advanced	Mid-therapy	CellSearch System	5 per 7.5 ml
4	Halle C.F. Moore, 2021 (37)	America	America	37	N/A	Advanced	Baseline	CellSearch System and HD-SCA assay	5 per 7.5 ml
5	Jeffrey B Smerage, 2014 (38)	America	America	288	N/A	Advanced	Post-therapy	CellSearch System	5 per 7.5 ml
6	Elisabeth Trapp, 2018 (39)	Germany	Europe	1087	53	Early	Post-therapy	CellSearch System	1 per 7.5 ml
7	Shunyun Pang, 2021 (40)	China	Asian	110	52.7	ALL	Baseline	Immunomagnetic nanospheres (IMNs)	19 per 7.5 ml
8	Markus Wallwiener, 2012 (41)	Germany	Europe	486	55	Advanced	Baseline	CellSearch System	5 per 7.5 ml
9	Carolyn S Hall, 2016 (42)	America	America	509	53	Early	Baseline	CellSearch System	1 per 7.5 ml
10	Jean-Marie Ramirez, 2014 (43)	Germany	Europe	254	60	Advanced	Baseline	EPISPOT and CellSearch system	1 per 7.5 ml
11	William Jacot, 2019 (44)	France	Europe	150	N/A	Advanced	Mid-therapy	CellSearch System	5 per 7.5 ml
12	Jean-Yves Pierga, 2015 (45)	France	Europe	52	50.6	Early	Baseline	CellSearch System	1 per 7.5 ml
13	Julia Jueckstock, 2016 (46)	Germany	Europe	1221	53	Early	Baseline	Manually performed immunocytochemistry	1 per 23 ml
14	Daniel F Hayes, 2006 (47)	America	America	177	N/A	Advanced	Baseline	CellSearch System	5 per 7.5 ml
15	Zhaomei Mu, 2015 (48)	America	America	115	54.5	Advanced	Baseline	CellSearch System	5 per 7.5 ml
16	Brigitte Rack, 2014 (12)	Germany	Europe	2026	N/A	Early	Post-therapy	CellSearch System	1 per 30 ml
17	Anna-Maria Larsson, 2018 (49)	Sweden	Europe	152	65	Advanced	Baseline	CellSearch System	5 per 7.5 ml
18	Yuqin Yang, 2022 (50)	China	Asia	216	46	Early	Mid-therapy	Liquid Biopsy System	1 per 4 ml
19	Yukako Shiomi-Mouri, 2013 (51)	Japan	Asia	97	59	Advanced	Baseline	CellSearch System	1 per 7.5 ml
20	Shaheenah Dawood, 2008 (52)	America	America	185	49	Advanced	Baseline	CellSearch System	5 per 7.5 ml
21	Mandar Karhade, 2014 (53)	America	America	105	54	Early	Baseline	CellSearch System	1 per 7.5 ml
22	Morales S, 2018 (54)	Spain	Europe	67	59.6	Advanced	Mid-therapy	CellSearch System and RT-PCR methods	5 per 7.5 ml
23	Naoki Hayashi, 2011 (55)	Japan	Asia	49	54.1	Advanced	Mid-therapy	CellSearch System	5 per 7.5 ml

**Table:** 

Outcome^e^	CTC status^d^		HR (95% CI)		Materials		Anatomic stage	Histologic grade	Lymph node involvement
	−	+	PFS/DFS	OS	Therapy methods^e^	Follow-up time^f^	I–IV	I–III	Y/N
DFS/OS	73	50	1.68 (0.85–3.32)	2.3 (0.95–5.57)	Accepted surgery	<20 months	33/67/23/0	1/18/101	52/71
PFS/OS	60	20	2.52 (1.4532–4.3704)	6.49 (2.1303–19.7735)	Systemic therapy	<20 months	N/A	N/A	N/A
PFS	239	138	1.22 (0.97–1.54)	N/A	Chemotherapy	>20 months	N/A	N/A	N/A
PFS	27	7	1.4 (0.59–3.32)	N/A	Accepted surgery	>20 months	N/A	N/A	N/A
PFS/OS	165	123	2.13 (1.63–2.79)	1.94 (1.52–2.47)	Chemotherapy	<20 months	IV	N/A	N/A
DFS/OS	889	198	1.37 (0.86–2.17)	2.07 (1.01–4.24)	Chemotherapy	>20 months	N/A	58/528/501	365/718
PFS/OS	55	55	3.56 (1.86–6.82)	4.98 (2.06–12.02)	Accepted surgery	>20 months	19/38/18/20	N/A	N/A
PFS/OS	281	205	1.82 (1.41–2.34)	4.79 (2.95–7.79)	Systemic therapy	<20 months	IV	N/A	N/A
RFS/OS	385	124	2.72 (1.57–4.72)	2.29 (1.12–4.67)	Accepted surgery	>20 months	I-III	56/232/202	234/273
OS	132	122	N/A	0.386 (0.223–0.668)	Chemotherapy	N/A	N/A	N/A	N/A
PFS/OS	64	86	2 (1.4–2.8)	3.6 (2.3–5.8)	Chemotherapy	<20 months	IV	N/A	N/A
DFS	34	18	3.69 (1.34–10.21)	N/A	Accepted surgery	>20 months	IV	N/A	N/A
DFS/OS	970	251	1.25 (0.88–1.77)	1.47 (0.96–2.23)	Accepted surgery	>20 months	ALL	59/604/557	1122/422
PFS/OS	90	87	1.89 (1.37–2.61)	2.45 (1.64–3.65)	Systemic therapy	>20 months	IV	N/A	N/A
PFS	79	36	2.38 (1.44–3.95)	N/A	Systemic therapy	<20 months	0/0/12/103	N/A	N/A
PFS/OS	1,174	330	2.257 (1.595–3.195)	2.447 (1.491–4.015)	Chemotherapy	>20 months	N/A	N/A	N/A
PFS/OS	73	79	1.68 (1.17–2.42)	2.52 (1.58–4.01)	Systemic therapy	>20 months	N/A	13/65/46	44/92
OS	172	44	N/A	1.934 (0.607–6.168)	Chemotherapy	<20 months	52/124/40/0	N/A	N/A
OS	53	45	N/A	3.816 (1.839–7.917)	Chemotherapy	N/A	N/A	10/42/28	N/A
OS	114	71	N/A	3.1 (1.8–5.2)	Chemotherapy	N/A	22/59/43/56	N/A	71/77
PFS/OS	71	34	3.93 (1.55–9.94)	2.36 (0.84–6.65)	Accepted surgery	>20 months	N/A	4/15/92	62/51
PFS	39	38	2.18 (1.22–3.9)	N/A	Systemic therapy	N/A	N/A	N/A	N/A
PFS/OS	28	21	2.627 (1.161–5.946)	3.096 (1.313–7.302)	Systemic therapy	>20 months	N/A	N/A	N/A

ref., reference; CTC, circulating tumor cell; CTC^+^, positive CTC detection; HR, hazard ratio; CI, confidence interval; OS, overall survival; PFS, progression-free survival; DFS, disease-free survival; RFS, relapse-free survival; N/A, not applicable; nMBC, non-metastatic breast cancer; MBC, metastasis breast cancer.

In order for the analysis to be successful:

a Except for the CellSearch System, other methods and combinations are deemed as “Not CellSearch System” in the meta-analysis.

b In addition to “1 per 7.5 ml” and “5 per 7.5 ml,” other CTC^+^ definitions are considered as “other CTC^+^” in the meta-analysis.

c Based on the previous studies, the DFS/PFS/RFS could be regarded as the same data to calculate HR, and single data are not declared in the analysis.

d The number of samples detected by the detection methods was not the same as the population involved in the corresponding clinical trials; the “–” label meant that the CTC could not be detected by the detection methods, and vice versa.

e The detailed therapy information classified by the subgroups includes chemotherapy, accepted surgery, and systemic therapy (surgery + other treatment).

f The follow-up time is divided into “<20 months” and “>20 months.” If the data could not be classified, it would be considered as “not applicable.”

### The diagnostic performance of CTC detection

**Figure 2:**
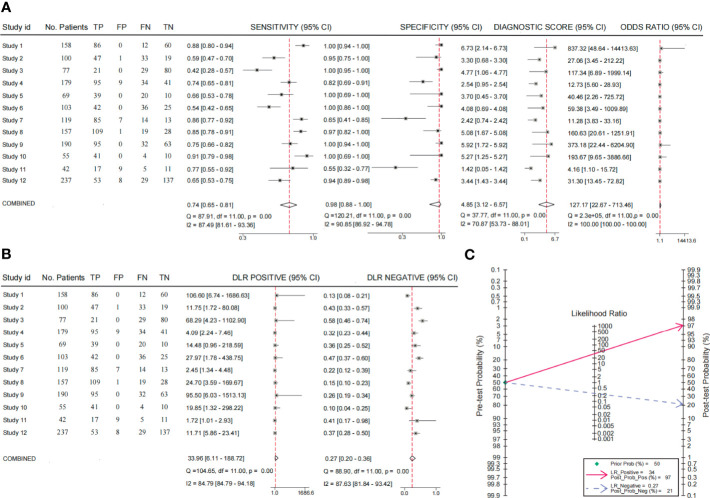
Analysis of the diagnostic values of CTC. **(A)** The sensitivity, specificity, diagnostic score, and odds ratio of CTCs for the diagnosis of BC; **(B)** the analysis of DLR positive and DLR negative; **(C)** the Fagan nomogram of the diagnostic values of CTCs.

Among the 12 included studies, 7 studies investigated the diagnostic efficacy of CTC detection for all cancer stages, 4 studies mainly focused on the metastatic stage, and 1 study did not provide information on the cancer stage. The results showed a high diagnostic efficacy of CTC detection with 0.74 sensitivity (95% CI, 0.65–0.81) and 0.98 specificity (95% CI, 0.88–1.00). The diagnostic score and odds ratio were 4.85 and 127.17, respectively (**Figure 2A**). Positive and negative diagnostic likelihood ratios (DLRs) were 33.96 and 0.27 (**Figure 2B**). Remarkably, the Fogan diagram indicated that an individual who tested positive with a CTC test had a 97% chance of developing BC (**Figure 2C**). This indicated that the detection methods had good effectiveness for CTCs. The summary receiver operating characteristic curves (SROCs) showed an area under the curve of 0.89 (95% CI, 0.86–0.91). Combined with the diagnostic odds ratio, the result also provided evidence of the values of those CTC detection methods (**Supplementary Figures S1A, B**). Heterogeneity was significant in these analyses (*I* ^2^ > 50%). However, the funnel plot asymmetry test with linear regression indicated a non-significant publication bias in the meta-analysis (*P* = 0.37) (**Supplementary Figure S1C**). Thus, we performed a metaregression analysis and showed that continent was the potential source of heterogeneity. Our subgroup analysis indicated that specificity would be higher in Chinese patients (**Supplementary Table 7**).

### Factors that influence the association between CTC and poor prognosis

In order to identify the factors that influence the CTC^+^ prognosis value, the variables were examined in the metaregression, including publication year, sample size, age, continent, detection method, CTC^+^ definition, tumor stage, therapeutic regimen, sampling time, and follow-up time (**Supplementary Table 8**). Our results showed that the detection method and continent were the major elements of the heterogeneity in the pooled HR_OS_ and HR_PFS/DFS_ (*P* = 0.01). Then, we divided the subgroups and analyzed them according to the differences in the detection method and continent.

**Figure 3:**
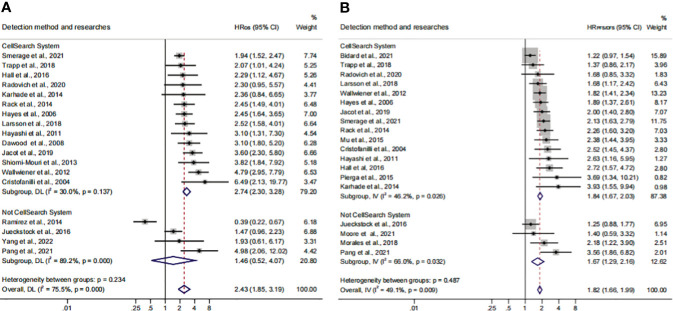
The subgroup analysis of detection methods in prognosis value. **(A)** The pooled HR_OS_ of the detection method; **(B)** the pooled HR_PFS/DFS_ of the detection method.

Previous studies demonstrated that the CellSearch System was the most used detection system for CTC detection (56). More recently, researchers have combined two systems/methods for detection, namely, immunomagnetic nanospheres (IMNs) and reverse transcription-polymerase chain reaction (RT-PCR), to improve the significance of the CTC prognostic value. Those studies were classified and analyzed as another subset in the subgroup analysis, which was named the Not CellSearch System subset (**Figure 3**). The calculated analysis revealed that CTC^+^ was associated with poor survival and could be regarded as a high-risk biomarker (HR_OS_, 2.43; 95% CI, 1.85–3.19; HR_PFS/DFS_, 1.82; 95% CI, 1.66–1.99) (**Figures 3A, B**; **Table 3**). The overall heterogeneity was significant in the OS analysis (*I* ^2 ^= 75.5%). We suspected that heterogeneity might come from the Not CellSearch System subset (*I* ^2 ^= 89.2%); however, publication bias did not exist in this subset (*P*_Begg_ > 0.05; *P*_Egger_ = 0.652) (**Supplementary Figures S2A, B**; **Table 4**). The one-way sensitivity analysis considered that the exclusion of any article did not affect the entire outcome (**Supplementary Figure S2C**). The trim-and-fill analysis suggested that one study might be missed and that if it were published, the relationship would not be reversed (the adjusted HR, 0.99; 95% CI, 0.351–2.724, **Supplementary Figure S2D**; **Table 3**). Furthermore, the association between CTC^+^ and poor OS would be obvious if the CellSearch System was utilized as the detection system in the clinical trial (HR = 2.74; 95% CI, 2.30–3.28).

**Table 3:** Summary of subgroup meta-analysis for CTC prognosis value evaluation.

Subgroup		HR_OS_ (95% CI)	HR_PFS/DFS_ (95% CI)
Detection method and research	CellSearch System	2.74 (2.30, 3.28)	1.84 (1.67, 2.03)
	Not CellSearch System	1.46 (0.52, 4.07)	1.67 (1.29, 2.16)
CTC and research	1 per 7.5ml	1.79 (0.81, 3.93)	2.04 (1.53, 2.72)
	5 per 7.5ml	2.96 (2.27, 3.87)	1.78 (1.60, 1.98)
	Other CTC definition	2.26 (1.38, 3.71)	1.85 (1.47, 2.33)
Continent and research	Europe	2.00 (1.12, 3.58)	1.62 (1.44, 1.82)
	America	2.31 (1.90, 2.81)	2.15 (1.83, 2.52)
	Asian	3.51 (2.27, 5.42)	3.16 (1.90, 5.26)
Therapy and research	Chemotherapy	2.04 (1.27, 3.30)	1.69 (1.47, 1.93)
	Accepted surgery	2.23 (1.47, 3.40)	1.91 (1.52, 2.39)
	Systemic therapy	3.23 (2.30, 4.54)	1.95 (1.67, 2.26)
Sample time and research	Baseline	2.31 (1.46, 3.67)	1.88 (1.64, 2.16)
	Mid-therapy	3.09 (2.17, 4.39)	1.53 (1.29, 1.82)
	Post-therapy	2.29 (1.65, 3.18)	2.06 (1.72, 2.47)
Tumor stage and research	Advanced stage	2.57 (1.70, 3.88)	1.78 (1.60, 1.98)
	Early stage	1.97 (1.54, 2.52)	1.83 (1.52, 2.20)
All research studies	–	2.43 (1.85, 3.19)	1.82 (1.66, 1.99)

**Table 4:** Summary of the bias analysis and trim-and-fill analysis.

Factors	OS					PFS/DFS				
	Publication bias			The adjusted HR of trim-and-fill analysis			Publication bias		The adjusted HR of trim-and-fill analysis	
	Subset	Begg’s funnel	Egger’s funnel	Missed studies	Adjusted HR	Subset	Begg’s funnel	Egger’s funnel	Missed studies	Adjusted HR
Detection system	Not CellSearch System	1	0.64	1	0.98 (0.35–2.72)	Not CellSearch System	0.50	0.40	1	1.51 (0.87–2.61)
Continent	Europe	0.45	0.66	2	1.51 (0.84–2.72)	Europe	0.35	0.60	3	1.51 (1.36–1.68)
CTC definition	1 per 7.5 m	0.85	0.11	2	1.25 (0.61–2.58)	1 per 7.5 m	0.60	0.54	1	1.68 (0.99–2.87)
	5 per 7.5 m	0.08	0.02	5	2.17 (1.64–2.88)	5 per 7.5 m				
	Other	0.50	0.42	2	1.55 (0.90–2.66)	Other				
Therapeutic plan	Chemotherapy	0.80	0.95	3	1.57 (1.02–2.41)	Chemotherapy	1	0.58	0	1.74 (1.33–2.29)
						Accepted surgery	0.65	0.09	2	1.87 (1.28–2.74)
Sample time	Baseline	0.53	0.69	3	1.78 (1.15–2.77)	Mid-therapy	0.62	0.13	2	1.50 (1.13–2.01)
Cancer stage	Advanced stage	1	0.65	1	0.98 (0.35–2.72)	Early stage	0.29	0.19	2	1.75 (1.28–2.39)

**Figure 4:**
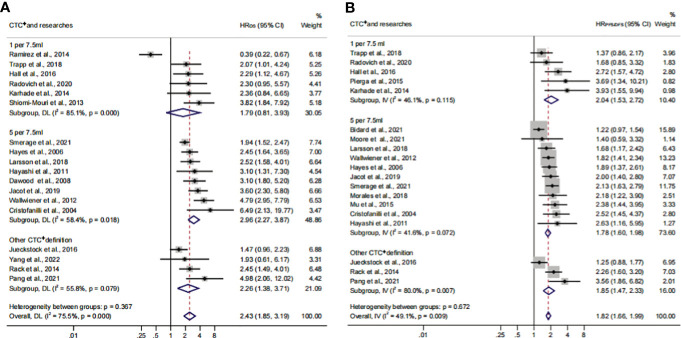
The subgroup analysis of CTC^+^ definition in prognosis value. **(A)** The calculated HR_OS_ of CTC^+^ definition; **(B)** the calculated HR_PFS/DFS_ of CTC^+^ definition.

The different detection methods implied that variations existed in the threshold levels. Thus, we performed a subgroup analysis for the CTC^+^ definition according to the different threshold levels. The pooled results suggested that CTC^+^ was a stable prognosticator in poor survival assessment (HR_OS_, 2.43; 95% CI, 1.85–3.19; HR_PFS/DFS_, 1.82; 95% CI, 1.66–1.99) (**Figures 4A, B**; **Table 3**). The overall heterogeneity was significant in the PFS/DFS subgroup (*I* ^2 ^= 75.5%). The publication bias, one-way sensitivity, and the trim-and-fill analysis demonstrated that the results were reliable and not reversed (the adjusted HR, 1.68) (**Supplementary Figure S2E–H**; **Table 4**). When the CTC^+^ was defined as 1 CTC per 7.5 mL, the poor PFS/DFS was significantly associated with CTC^+^ (HR, 2.04; 95% CI, 1.53–2.72). Meanwhile, the CTC^+^ was defined as 5 CTCs per 7.5 mL, and the poor OS was more significantly associated with CTC^+^ (HR_OS_, 2.96; 95% CI, 2.27–3.87).

### Association of CTC^+^ prognostic value in different continents

**Figure 5:**
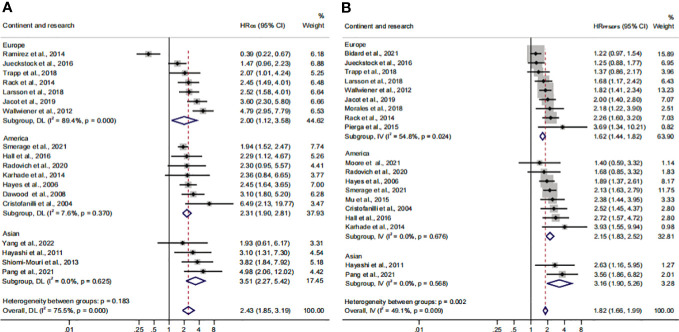
The continent subgroup analysis of prognosis assessment. **(A)** The pooled HR_OS_ of continent; **(B)** the pooled HR_PFS/DFS_ of continent.

To identify another source of heterogeneity, we conducted a subgroup analysis, based on the difference of continents (**Figure 5**; **Table 3**). Our results showed that the relationship of CTC^+^ with poor survival was not influenced by the different regions (HR_OS_ = 2.43; 95% CI, 1.85–3.19; HR_PFS/DFS_ = 1.82; 95% CI, 1.66–1.99) (**Figures 5A, B**; **Table 3**). The heterogeneity was moderate in the PFS/DFS subgroup (*I* ^2 ^= 49.1%). Furthermore, the heterogeneity might mainly come from the Europe subset (*I* ^2 ^= 54.8%). However, publication bias did not exist (**Supplementary Figures S2I, J**; **Table 4**). The outcome was not changed in the one-way sensitivity analysis (**Supplementary Figure S2K**). The adjusted HR would be 1.512 after the trim-and-fill analysis (95% CI, 1.358–1.682) (**Supplementary Figure S2L**; **Table 4**). Compared with the subsets in the subgroup analyses, CTC^+^ may be closely related to worse survival in Asian patients (HR_OS_, 3.51; 95% CI, 2.27–5.42; HR_PFS/DFS_, 3.16; 95% CI, 1.90–5.26).

### Relationship between CTC+ prognostic value and clinical therapeutic characteristics

**Figure 6:**
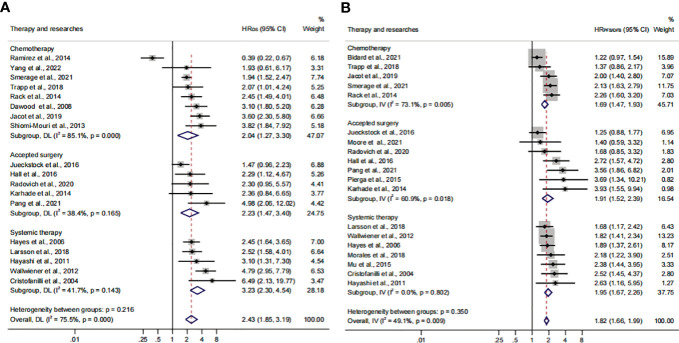
The therapy subgroup analysis of survival evaluation. **(A)** The calculated HR_OS_ of therapy; **(B)** the calculated HR_PFS/DFS_ of therapy.

Previous studies considered that some drugs, such as sorafenib and digitoxin, could limit or kill tumor cells detaching from the primary distant sites (57). Thus, we conducted a subgroup analysis to investigate the influence of different treatment methods and sampling times on the prognostic value of CTC^+^. The calculated HR suggested that the relationship was not affected by different treatment methods (HR_OS_, 2.43; 95% CI, 1.85–3.19; HR_PFS/DFS_, 1.82; 95% CI, 1.66–1.99) (**Figures 6A, B**; **Table 3**). The heterogeneity was significant in the OS subgroup analysis (*I* ^2 ^= 75.5%). Comparing the heterogeneity of the subsets, the results indicated that the source came from the chemotherapy subset (*I* ^2 ^= 85.1%). However, publication bias was not discovered in this subset (**Supplementary Figures S2M, N**). The outcome was stable in the one-way sensitivity analysis (**Supplementary Figure S2O**). The adjusted HR would be 1.57 analyzed by the trim-and-fill analysis (**Supplementary Figure S2P**; **Table 4**). Furthermore, the subset results showed that the patients who received systemic therapy would have worse survival than other patients when the CTC was detected (HR_OS_, 3.23; 95% CI, 2.30–4.45; HR_PFS/DFS_, 1.95; 95% CI, 1.67–2.26).

**Figure 7:**
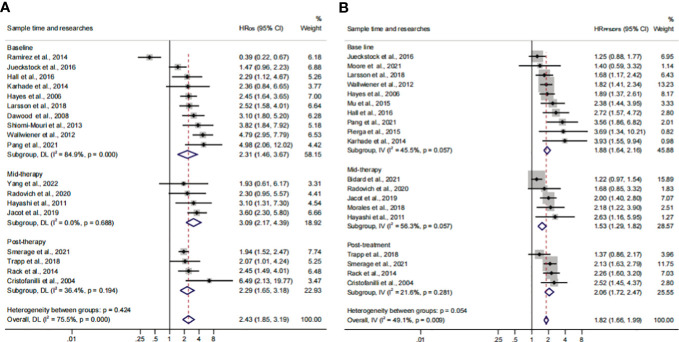
The sample time and therapy subgroup analysis of survival evaluation. **(A)** The pooled HR_OS_ of sample time; **(B)** the pooled HR_PFS/DFS_ of sample time.

The subgroup analysis of sampling times indicated that the relationship between CTC^+^ and poor survival would be stable regardless of the treatment phases (HR_OS_, 2.43; 95% CI, 1.85–3.19; HR_PFS/DFS_, 1.82; 95% CI, 1.66–1.99) (**Figures 7A, B**; **Table 3**). The heterogeneity of OS analysis was significant (*I* ^2 ^= 75.5%), and the heterogeneity of the subgroup might result from the baseline subset (*I* ^2 ^= 84.9%). The result was reliable after the publication bias, the one-way sensitivity, and the trim-and-fill analysis (**Supplementary Figures S3A–D**; **Table 4**). Consistent with a previous study, our result showed that CTC detection at mid-therapy or post-therapy could be used for monitoring therapeutic effects and had prognostic relevance (58). For instance, the subset outcomes exhibited that patients with CTC^+^ would have worse OS and PFS/DFS in both mid-therapy and post-therapy (HR_OS_, 3.09; 95% CI, 2.17–4.39; HR_PFS/DFS_, 2.06, 95% CI, 1.72–2.47).

### The analysis of CTC prognosis value for all patients and cancer stages

**Figure 8:**
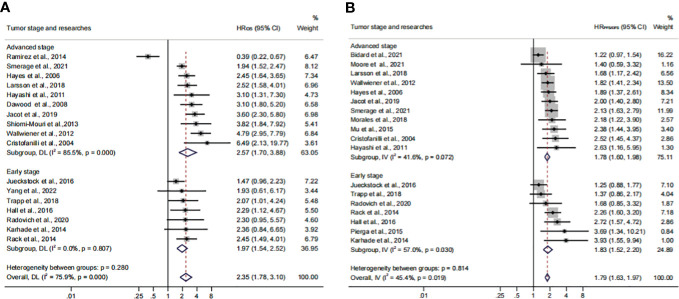
Analysis of prognosis values in different stages. **(A)** The calculated HR_OS_ of the tumor stage; **(B)** the calculated HR_PFS/DFS_ of the tumor stage.

In order to investigate the prognostic value of CTC**^+^** in different stages of BC, we conducted a subgroup analysis. We divided the data into subgroups according to cancer stages. The calculated analysis showed that the prognostic value of CTCs would not be affected by the cancer stages (HR_OS_, 2.35; 95% CI, 1.78–3.10; HR_PFS/DFS_, 1.79; 95% CI, 1.63–1.97) (**Figures 8A, B**; **Table 3**). The OS subgroup analysis showed a significant heterogeneity (*I* ^2 ^= 75.9%), and it might come from the advanced stage subset (*I* ^2 ^= 85.5%). However, the result was stable after the publication bias, the one-way sensitivity, and the trim-and-fill analysis (**Supplementary Figures S4E–H**; **Table 4**). Furthermore, the relationship between CTC and poor OS was more obvious in the advanced BC stage (HR_OS_, 2.57; 95% CI, 1.70–3.88). However, as for the poor PFS/DFS forecast, early BC stage patients may benefit more from the relationship (HR_PFS/DFS_, 1.83; 95% CI, 1.52–2.20).

**Figure 9:**
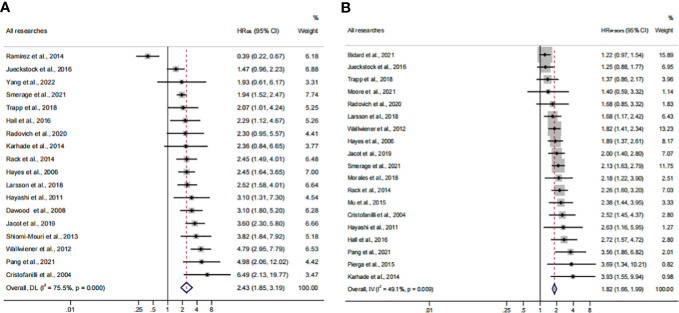
Analysis of prognosis values for all patients. **(A)** The pooled HR_OS_ of all research studies; **(B)** the pooled HR_PFS/DFS_ of all research studies.

Herein, 18 available trials and 6,794 individuals could be unitized in the HR_OS_ extraction. HR_PFS/DFS_ was available in 19 studies, which consisted of 6,696 patients. The analyzed HR indicated that CTC^+^ could represent poor survival in all BC patients (HR_OS_, 2.43; 95% CI, 1.85–3.19; HR_PFS/DFS_, 1.82; 95% CI, 1.66–1.99) (**Figures 9A, B**; **Table 3**). The heterogeneity was significant in all BC analyses; however, the metaregression and subgroup analyses showed that the result was stable and reliable. Despite the overall heterogeneity being moderate in some subgroups (*I* ^2^ < 50%) (**Figures 3B**, **4B**, **5B**, **6B**, **7B**, **8B**), the heterogeneity analyses of the subsets in the subgroup analysis could support the stability and reliability of the analyses (*I* ^2^ > 50%) (**Supplementary Figures S3E–P**, **S4A–D, I–P**, **S5**; **Table 4**).

## Discussion

In a rapidly evolving cancer prediction field, CTC detection technologies have attracted the attention of researchers. In this study, we investigated the diagnostic effectiveness of the widely used CTC detection approaches including the CellSearch System, ICC, and RT-PCR. Our results suggested that CTC detection was effective and had high diagnostic value for BC patients. The CellSearch System might have a higher diagnostic value compared with other detection methods. Moreover, the different threshold levels do not affect the relationship between CTC^+^ and poor prognosis. In the prognosis subanalysis, patients detected by the CellSearch System with CTC**^+^** showed a worse prognosis (HR_OS_, 2.74; 95% CI, 2.30–3.28; HR_PFS/DFS_, 1.84, 95% CI, 1.67–2.03). The subgroup analysis for patients from continents indicated that CTC^+^ was associated with a worse OS and PFS/DFS in the Asian population which was consistent with a previous study and could be regarded as a more obvious biomarker for patients from Asia (16, 59, 60). It may be caused by the differences between ethnic, but the specific reason is not yet clear and needs further research.

Most studies revealed that the presence of CTCs implied a worse prognosis at baseline. However, the relationships between CTCs and the therapeutic regimen were unclear. Our results demonstrated that CTC**^+^** at mid-/post-therapy could not only reflect the therapeutic effect but also evaluate the prognostic relevance. The prognostic ability was not influenced by the time points of sampling. Moreover, our research also showed that the relationship between CTC**^+^** and poor survival was changing constantly. Thus, we suggest that patients should repeat the CTC detection after the therapy to obtain a more accurate survival assessment. Comparing the three time points, the pooled HRs indicated that patients should conduct CTC detection again after treatment to obtain a more accurate evaluation of survival. Altogether, the association between CTCs and poor survival should be considered stable, regardless of the treatment methods the patient is receiving. However, the relationship between CTC**^+^** and different therapeutic regimens was not investigated in the subgroup analysis due to insufficient information which deserves further study in the future. Previous studies have demonstrated that the prognosis of early and advanced BC was obviously different (61, 62). In line with previous studies, our findings showed that CTC^+^ could serve as an independent predictor for cancer progression (35, 63–65). To sum up, our study showed that CTCs could be utilized as a high-value marker for all BC patients. The value was stable after the heterogeneity analysis. Moreover, CTC detection should be conducted in different stages which could predict the prognosis and treatment response for BC patients.

With the development of detection techniques, novel methods such as microgels and antifouling nanofilm could facilitate in the separation and purification of CTCs, which could promote the CTCs to enter the clinic (66–68). Although the utility of CTC detection was not included in the clinical practice guidelines of BC, many studies have shown its great potential in the management of BC patients (69, 70). For instance, the results obtained from Chakraborty’s group encouraged the incorporation of CTC quantification as a prognostic marker and for minimally invasive tumor burden assessment in multiple myeloma (71). In the future, the level of CTCs might be an important component of stage definition for BC patients. Moreover, investigation of the genomic/transcriptional/proteomic profiles of CTCs could provide comprehensive information in choosing therapeutic strategies. For example, some CTC measurement technologies achieved the genotyping of CTCs, including crucial gene mutations and clone heterogeneity, such as TP53, PIK3CA, ERBB2, KLK10, NUMBL, GFB1, and BSG (72, 73). Those achievements would help clinicians select personalized treatment and more effective therapeutic regimens during tumor progression. The present studies suggested the clinical value of CTCs in the diagnosis, prognosis, and treatment of BC. However, the utilization of CTCs urgently needs standard detection methods and clinical guidelines, especially for the differences in populations, therapeutic schemes, BC stages, thresholds, and the appropriate time points for blood sampling (74).

CTCs could not be the unique prognostic factor due to the complex mechanism of BC development, invasion, and metastasis. The progression of BC could be regulated by the tumor microenvironment (TME) and deeply influenced by cancer-associated fibroblasts, macrophages, neutrophils, T regulatory cells, tumor-infiltrating lymphocytes, and the related secreted molecules (75). For example, the number of CD68^+^ macrophages, the count of tumor-infiltrating lymphocytes, and the expression of TGF-β in different genetic levels could serve as prognostic and predictive markers (76–78). Those specific cells and related secreted molecules were equally important for the evaluation of BC prognosis. The design and construct of drugs aimed at those molecules would be a promising way for BC patients. For instance, Yi and his team produced bispecific antibodies targeting TGF-β and human PD-L1 (termed YM101 and BiTP) showing antitumor activity in the TNBC. This means that CTCs, as a kind of TME-related molecule, would also have the potential possibility to be utilized by the drug design of BC (79, 80).

Some limitations exist and should be considered deliberately. First, compared with other cancers, BC was relatively general. Meanwhile, some confounding factors were not clear and discussed which are equally important including detection markers, anatomic stages, histologic grades, and the metastatic conditions of the lymph nodes/organs. These factors could not be regarded as subgroups. Second, only a fraction of the literature directly offered univariate HR, LCI, and UCI values. In order to ensure the accuracy of data, studies were not included in this analysis that do not provide original data. Some articles only exhibited survival curves. These articles were not included because extracting data from survival curves also led to measurement bias. Third, according to the funnel plot, the meta-analysis adopted a systematic retrieval strategy and did not identify significant publication bias. However, some gray publications were not taken into analysis factually including meetings and abstracts written in other languages and inaccessible articles. Fourth, a series of data were still not detailed enough in the analysis. For example, the first follow-up time and the definition of BC stages in some articles were not clear. This could also influence the analysis outcomes. Finally, in our analysis, only one male patient was involved. This meant that the final outcome may be not valuable for men.

## Conclusions

Our results provided the latest evidence to support that CTCs have a high and stable value of the diagnosis and prognosis for BC, especially for patients from Asia. We suggest that patients should have CTC detection sequentially during treatment, especially when BC progression has been identified. In the future, novel techniques should be developed to improve the efficacy of CTC detection.

## Author contributions

HZ: Investigation, Methodology, Writing – original draft. LW: Investigation, Methodology, Writing – original draft. CF: Conceptualization, Writing – review & editing. CL: Conceptualization, Funding acquisition, Writing – review & editing. LZ: Conceptualization, Funding acquisition, Investigation, Writing – review & editing.

## Supplementary material

Supplementary Figure 1 The analyses of SROC with prediction & confidence contours, diagnostic odds ratio and Deeks’ funnel plot asymmetry Test in the diagnosis effect of CTC.

Supplementary Figure 2 The further identification and analysis of the heterogeneity. **(A)** the Begg’s funnel plot of the ‘Not CellSearch system’ group analysis in HR_OS_; **(B)** the Egger’s publication bias plot of the ‘Not CellSearch system’ group analysis in HR_OS_; **(C)** the one-way sensitivity analysis of the ‘Not CellSearch system’ group analysis in HR_OS_; **(D)** the trim-and-fill analysis of the ‘Not CellSearch system’ group analysis in HR_OS_; **(E)** the Begg’s funnel plot of ‘other definition of the CTC+’ group analysis in HR_PFS/DFS_; **(F)** the Egger’s publication bias plot of the ‘other definition of CTC+’ group analysis in HR_PFS/DFS_; **(G)**the one-way sensitivity analysis of ‘other definition of the CTC+’ group analysis in HR_PFS/DFS_; **(H)** the trim-and-fill analysis of the ‘other definition of CTC+’ group analysis in HR_PFS/DFS_; **(I)** the Begg’s funnel plot of the ‘Europe’ group analysis in HR_PFS/DFS_; **(G)** the Egger’s publication bias plot of the ‘Europe’ group analysis in HR_PFS/DFS_;**(K)** the one-way sensitivity analysis of the ‘Europe’ group analysis in HR_PFS/DFS_; **(L)** the trim-and-fill analysis of the ‘Europe’ group analysis in HR_PFS/DFS_; **(M)** the Begg’s funnel plot of the ‘chemotherapy’ group analysis in HR_OS_; **(N)** the Egger’s publication bias plot of the ‘chemotherapy’ group analysis in HR_OS_; **(O)** the one-way sensitivity analysis of the ‘chemotherapy’ group analysis in HR_OS_; **(P)** the trim-and-fill analysis of the ‘chemotherapy’ group analysis in HR_OS_.

Supplementary Figure 3 The further identification and analysis of the heterogeneity. **(A)** the Begg’s funnel plot of the ‘baseline’ group analysis in HR_OS_; **(B)** the Egger’s publication bias plot of the ‘baseline’ group analysis in HR_OS_; **(C)** the one-way sensitivity analysis of the ‘baseline’ group analysis in HR_OS_; **(D)** the trim-and-fill analysis of the ‘baseline’ group analysis in HR_OS_; **(E)** the Begg’s funnel plot of the ‘Europe’ group analysis in HR_OS_; **(F)** the Egger’s publication bias plot of the ‘Europe’ group analysis in HR_OS_; **(G)** the one-way sensitivity analysis of the ‘Europe’ group analysis in HR_OS_; **(H)** the trim-and-fill analysis of the ‘Europe’ group analysis in HR_OS_; **(I)** the Begg’s funnel plot of the ‘1 per 7.5 ml’ group analysis in HR_OS_; **(G)** the Egger’s publication bias plot of the ‘1 per 7.5 ml’ group analysis in HR_OS_;**(K)** the one-way sensitivity analysis of the ‘1 per 7.5 ml’ group analysis in HR_OS_; **(L)** the trim-and-fill analysis of the ‘1 per 7.5 ml’ group analysis in HR_OS_; **(M)** the Begg’s funnel plot of the ‘5 per 7.5 ml’ group analysis in HR_OS_; **(N)** the Egger’s publication bias plot of the ‘5 per 7.5 ml’ group analysis in HR_OS_; **(O)** the one-way sensitivity analysis of the ‘5 per 7.5 ml’ group analysis in HR_OS_; **(P)** the trim-and-fill analysis of the ‘5 per 7.5 ml’ group analysis in HR_OS_.

Supplementary Figure 4 The further identification and analysis of the heterogeneity. **(A)** the Begg’s funnel plot of the ‘other definition of CTC+’ group analysis in HR_OS_; **(B)** the Egger’s publication bias plot of the ‘other definition of CTC+’ group analysis in HR_OS_; **(C)** the one-way sensitivity analysis of the ‘other definition of CTC+’ group analysis in HR_OS_; **(D)** the trim-and-fill analysis of the ‘other definition of CTC+’ group analysis in HR_OS_; **(E)** the Begg’s funnel plot of the ‘advanced stage’ group analysis in HR_OS_; **(F)** the Egger’s publication bias plot of the ‘advanced stage’ group analysis in HR_OS_; **(G)** the one-way sensitivity analysis of the ‘advanced stage’ group analysis in HR_OS_; **(H)** the trim-and-fill analysis of the ‘advanced stage’ group analysis in HR_OS_; **(I)** the Begg’s funnel plot of the ‘Not CellSearch system’ group analysis in HR_PFS/DFS_; **(G)** the Egger’s publication bias plot of the ‘Not CellSearch system’ group analysis in HR_PFS/DFS_; **(K)** the one-way sensitivity analysis of the ‘Not CellSearch system’ group analysis in HR_PFS/DFS_; **(L)** the trim-and-fill analysis of the ‘Not CellSearch system’ group analysis in HR_PFS/DFS_; **(M)** the Begg’s funnel plot of the ‘mid-therapy’ group analysis in HR_PFS/DFS_; **(N)** the Egger’s publication bias plot of the ‘mid-therapy’ group analysis in HR_PFS/DFS_; **(O)** the one-way sensitivity analysis of the ‘mid-therapy’ group analysis in HR_PFS/DFS_; **(P)** the trim-and-fill analysis of the ‘mid-therapy’ group analysis in HR_PFS/DFS_.

Supplementary Figure 5 The further identification and analysis of the heterogeneity. **(A)** the Begg’s funnel plot of the ‘chemotherapy’ group analysis in HR_PFS/DFS_; **(B)** the Egger’s publication bias plot of the ‘chemotherapy’ group analysis in HR_PFS/DFS_; **(C)** the one-way sensitivity analysis of the ‘chemotherapy’ group analysis in HR_PFS/DFS_; **(D)** the trim-and-fill analysis of the ‘chemotherapy’ group analysis in HR_PFS/DFS_; **(E)** the Begg’s funnel plot of the ‘accepted surgery’ group analysis in HR_PFS/DFS_; **(F)** the Egger’s publication bias plot of the ‘accepted surgery’ group analysis in HR_PFS/DFS_; **(G)** the one-way sensitivity analysis of the ‘accepted surgery’ group analysis in HR_PFS/DFS_; **(H)** the trim-and-fill analysis of the ‘accepted surgery’ group analysis in HR_PFS/DFS_; **(I)** the Begg’s funnel plot of the ‘early stage’ group analysis in HR_PFS/DFS_; **(G)** the Egger’s publication bias plot of the ‘early stage’ group analysis in HR_PFS/DFS_; **(K)** the one-way sensitivity analysis of the ‘early stage’ group analysis in HR_PFS/DFS_; **(L)** the trim-and-fill analysis of the ‘early stage’ group analysis in HR_PFS/DFS._
