# Supplementary figures and images for: Factors influencing the diagnostic and prognostic values of circulating tumor cells in breast cancer: a meta-analysis of 8,935 patients
Source: Front Oncol. 2023 Nov 27;13:1272788. doi: 10.3389/fonc.2023.1272788 (PMC10711619; doi:10.3389/fonc.2023.1272788)

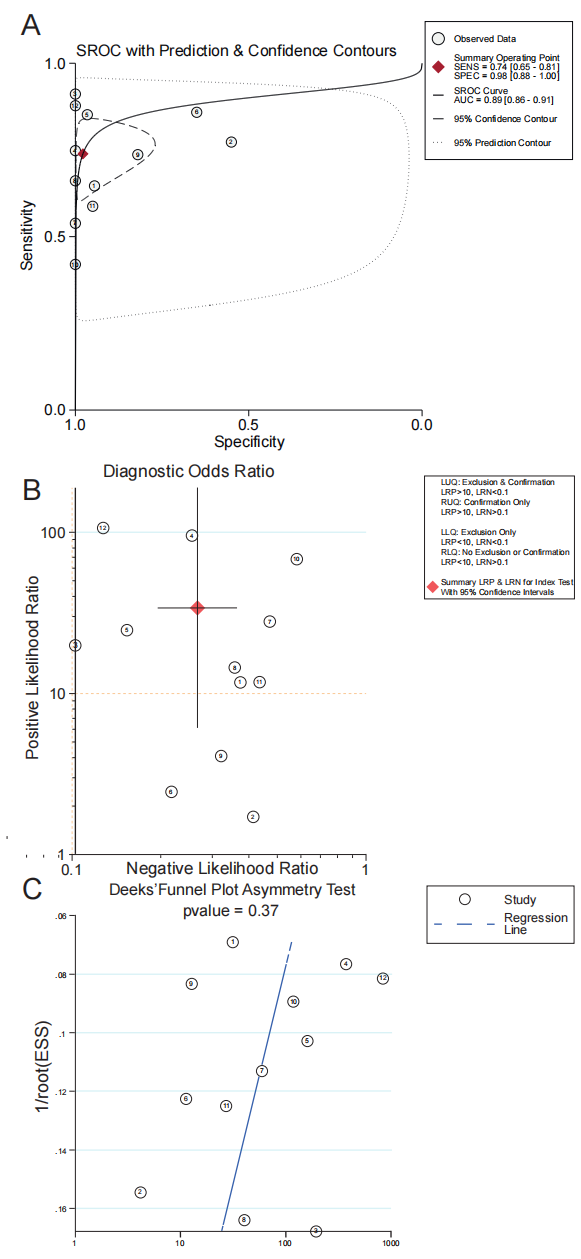

Supplement: Supplementary Figure 1 — The analyses of SROC with prediction & confidence contours, diagnostic odds ratio and Deeks’ funnel plot asymmetry Test in the diagnosis effect of CTC. [file Image_1.tif]

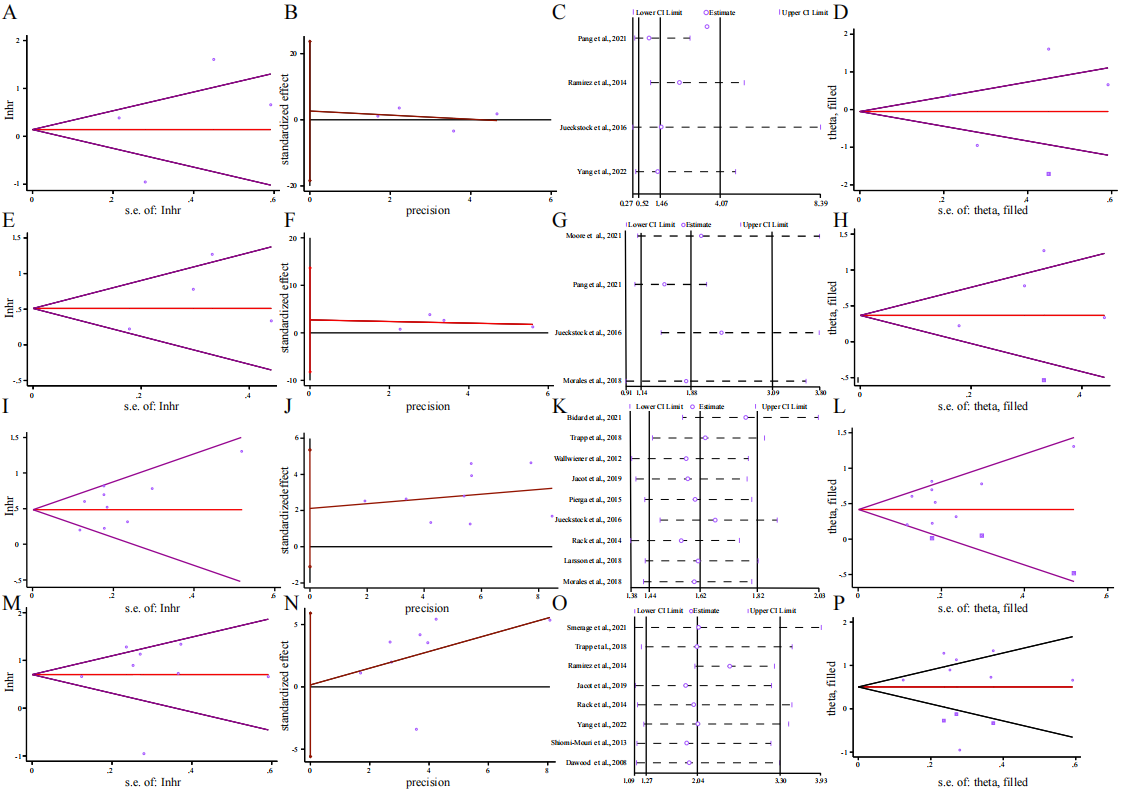

Supplement: Supplementary Figure 2 — The further identification and analysis of the heterogeneity. (A) the Begg’s funnel plot of the ‘Not CellSearch system’ group analysis in HROS; (B) the Egger’s publication bias plot of the ‘Not CellSearch system’ group analysis in HROS; (C) the one-way sensitivity analysis of the ‘Not CellSearch system’ group analysis in HROS; (D) the trim-and-fill analysis of the ‘Not CellSearch system’ group analysis in HROS; (E) the Begg’s funnel plot of ‘other definition of the CTC+’ group analysis in HRPFS/DFS; (F) the Egger’s publication bias plot of the ‘other definition of CTC+’ group analysis in HRPFS/DFS; (G)the one-way sensitivity analysis of ‘other definition of the CTC+’ group analysis in HRPFS/DFS; (H) the trim-and-fill analysis of the ‘other definition of CTC+’ group analysis in HRPFS/DFS; (I) the Begg’s funnel plot of the ‘Europe’ group analysis in HRPFS/DFS; (G) the Egger’s publication bias plot of the ‘Europe’ group analysis in HRPFS/DFS;(K) the one-way sensitivity analysis of the ‘Europe’ group analysis in HRPFS/DFS; (L) the trim-and-fill analysis of the ‘Europe’ group analysis in HRPFS/DFS; (M) the Begg’s funnel plot of the ‘chemotherapy’ group analysis in HROS; (N) the Egger’s publication bias plot of the ‘chemotherapy’ group analysis in HROS; (O) the one-way sensitivity analysis of the ‘chemotherapy’ group analysis in HROS; (P) the trim-and-fill analysis of the ‘chemotherapy’ group analysis in HROS. [file Image_2.tif]

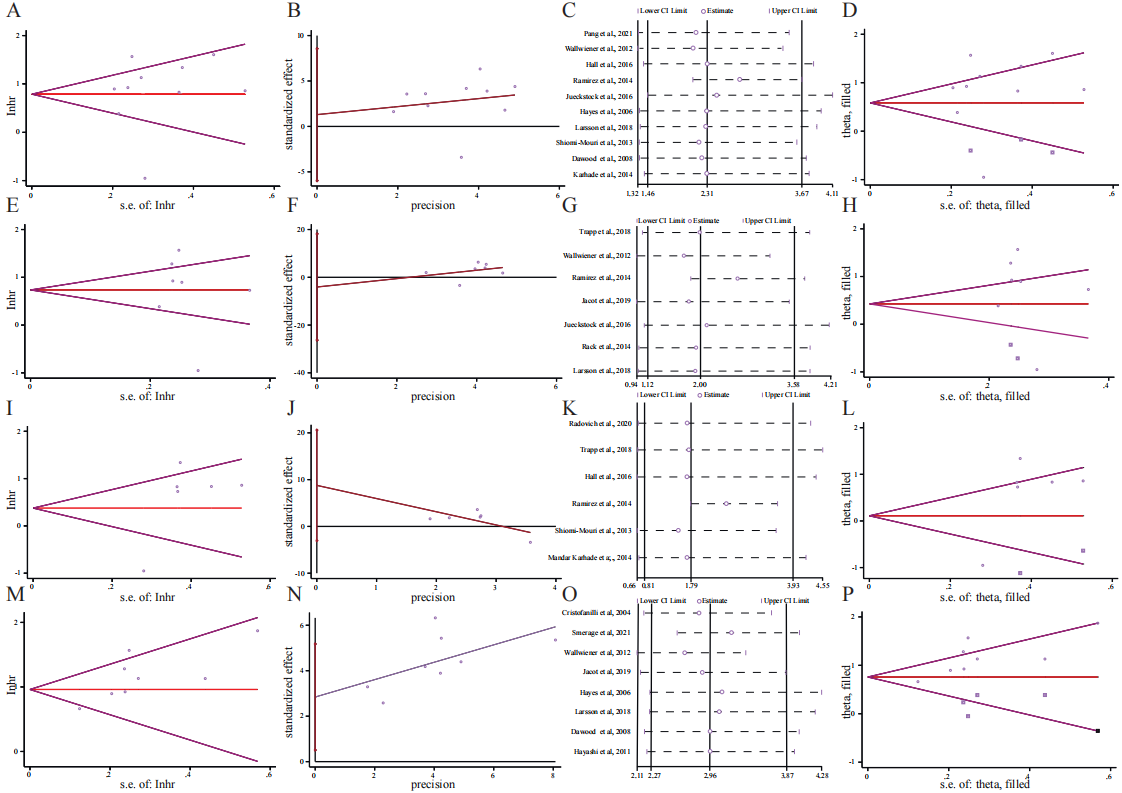

Supplement: Supplementary Figure 3 — The further identification and analysis of the heterogeneity. (A) the Begg’s funnel plot of the ‘baseline’ group analysis in HROS; (B) the Egger’s publication bias plot of the ‘baseline’ group analysis in HROS; (C) the one-way sensitivity analysis of the ‘baseline’ group analysis in HROS; (D) the trim-and-fill analysis of the ‘baseline’ group analysis in HROS; (E) the Begg’s funnel plot of the ‘Europe’ group analysis in HROS; (F) the Egger’s publication bias plot of the ‘Europe’ group analysis in HROS; (G) the one-way sensitivity analysis of the ‘Europe’ group analysis in HROS; (H) the trim-and-fill analysis of the ‘Europe’ group analysis in HROS; (I) the Begg’s funnel plot of the ‘1 per 7.5 ml’ group analysis in HROS; (G) the Egger’s publication bias plot of the ‘1 per 7.5 ml’ group analysis in HROS;(K) the one-way sensitivity analysis of the ‘1 per 7.5 ml’ group analysis in HROS; (L) the trim-and-fill analysis of the ‘1 per 7.5 ml’ group analysis in HROS; (M) the Begg’s funnel plot of the ‘5 per 7.5 ml’ group analysis in HROS; (N) the Egger’s publication bias plot of the ‘5 per 7.5 ml’ group analysis in HROS; (O) the one-way sensitivity analysis of the ‘5 per 7.5 ml’ group analysis in HROS; (P) the trim-and-fill analysis of the ‘5 per 7.5 ml’ group analysis in HROS. [file Image_3.tif]

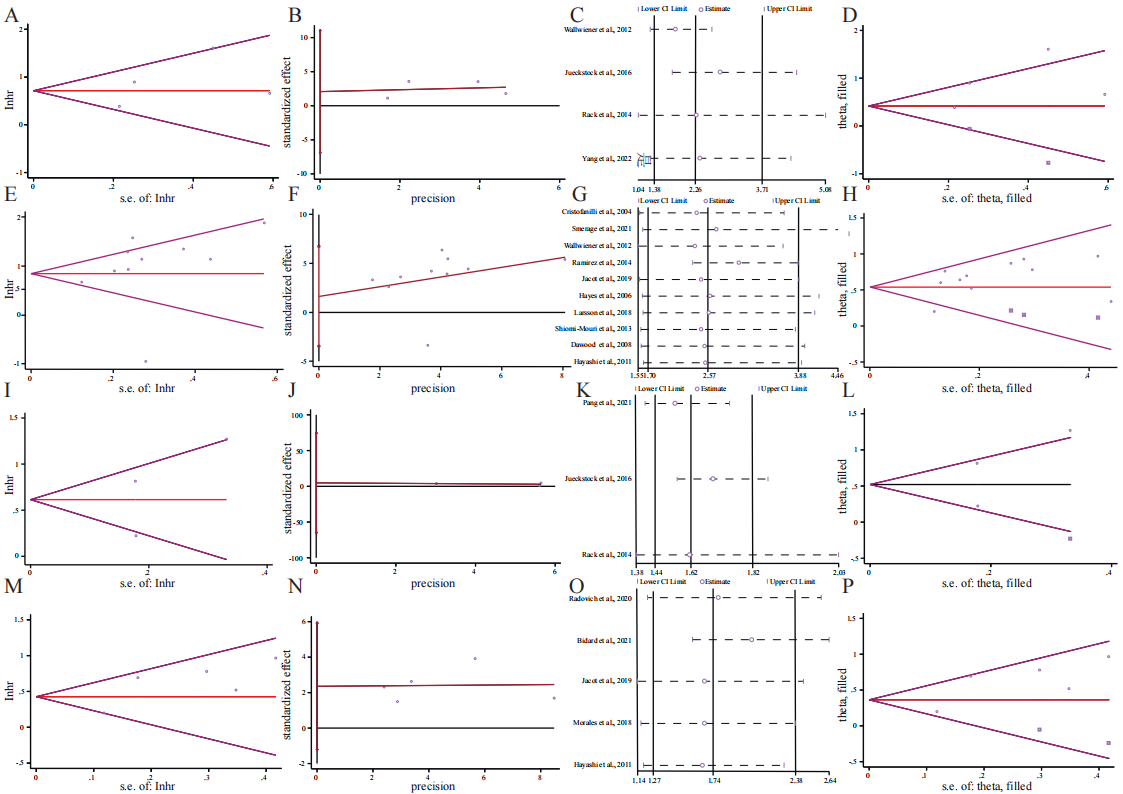

Supplement: Supplementary Figure 4 — The further identification and analysis of the heterogeneity. (A) the Begg’s funnel plot of the ‘other definition of CTC+’ group analysis in HROS; (B) the Egger’s publication bias plot of the ‘other definition of CTC+’ group analysis in HROS; (C) the one-way sensitivity analysis of the ‘other definition of CTC+’ group analysis in HROS; (D) the trim-and-fill analysis of the ‘other definition of CTC+’ group analysis in HROS; (E) the Begg’s funnel plot of the ‘advanced stage’ group analysis in HROS; (F) the Egger’s publication bias plot of the ‘advanced stage’ group analysis in HROS; (G) the one-way sensitivity analysis of the ‘advanced stage’ group analysis in HROS; (H) the trim-and-fill analysis of the ‘advanced stage’ group analysis in HROS; (I) the Begg’s funnel plot of the ‘Not CellSearch system’ group analysis in HRPFS/DFS; (G) the Egger’s publication bias plot of the ‘Not CellSearch system’ group analysis in HRPFS/DFS; (K) the one-way sensitivity analysis of the ‘Not CellSearch system’ group analysis in HRPFS/DFS; (L) the trim-and-fill analysis of the ‘Not CellSearch system’ group analysis in HRPFS/DFS; (M) the Begg’s funnel plot of the ‘mid-therapy’ group analysis in HRPFS/DFS; (N) the Egger’s publication bias plot of the ‘mid-therapy’ group analysis in HRPFS/DFS; (O) the one-way sensitivity analysis of the ‘mid-therapy’ group analysis in HRPFS/DFS; (P) the trim-and-fill analysis of the ‘mid-therapy’ group analysis in HRPFS/DFS. [file Image_4.tif]

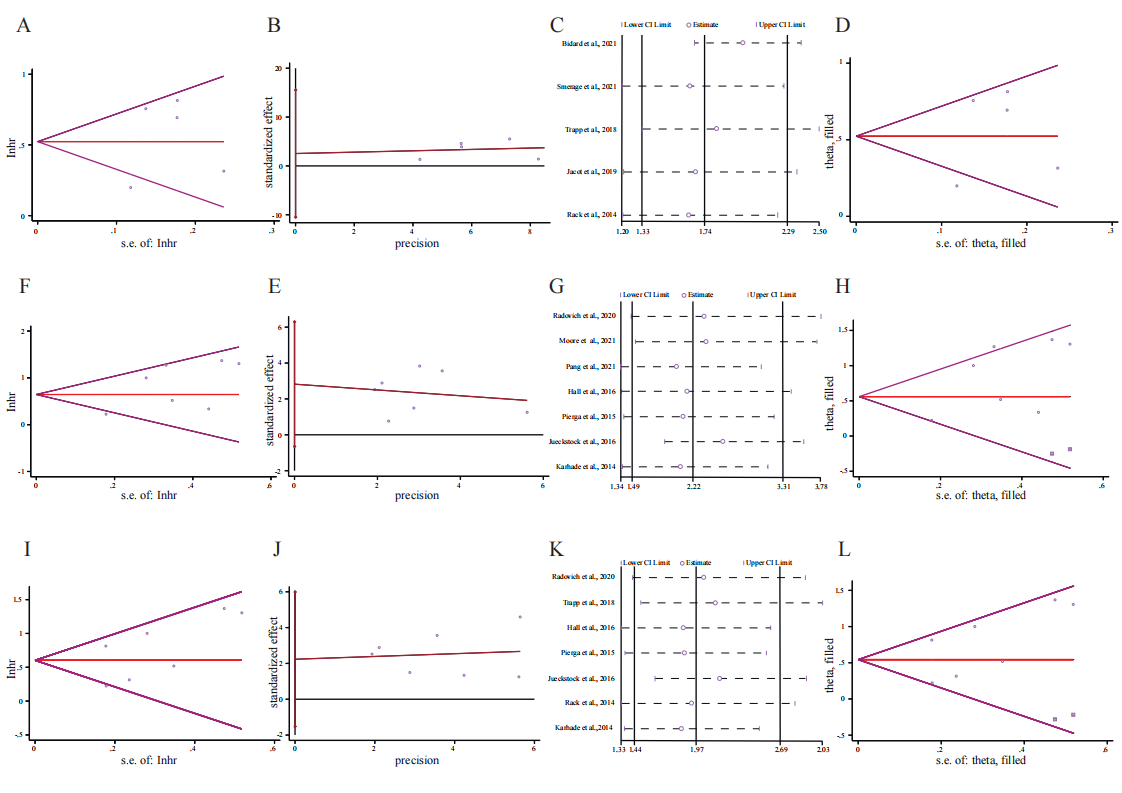

Supplement: Supplementary Figure 5 — The further identification and analysis of the heterogeneity. (A) the Begg’s funnel plot of the ‘chemotherapy’ group analysis in HRPFS/DFS; (B) the Egger’s publication bias plot of the ‘chemotherapy’ group analysis in HRPFS/DFS; (C) the one-way sensitivity analysis of the ‘chemotherapy’ group analysis in HRPFS/DFS; (D) the trim-and-fill analysis of the ‘chemotherapy’ group analysis in HRPFS/DFS; (E) the Begg’s funnel plot of the ‘accepted surgery’ group analysis in HRPFS/DFS; (F) the Egger’s publication bias plot of the ‘accepted surgery’ group analysis in HRPFS/DFS; (G) the one-way sensitivity analysis of the ‘accepted surgery’ group analysis in HRPFS/DFS; (H) the trim-and-fill analysis of the ‘accepted surgery’ group analysis in HRPFS/DFS; (I) the Begg’s funnel plot of the ‘early stage’ group analysis in HRPFS/DFS; (G) the Egger’s publication bias plot of the ‘early stage’ group analysis in HRPFS/DFS; (K) the one-way sensitivity analysis of the ‘early stage’ group analysis in HRPFS/DFS; (L) the trim-and-fill analysis of the ‘early stage’ group analysis in HRPFS/DFS. [file Image_5.tif]
