# Supplementary material for: Factors influencing the diagnostic and prognostic values of circulating tumor cells in breast cancer: a meta-analysis of 8,935 patients
Source: Front Oncol. 2023 Nov 27;13:1272788. doi: 10.3389/fonc.2023.1272788 (PMC10711619; doi:10.3389/fonc.2023.1272788)
Supplement: Supplementary file 8 [file Table_3.docx]

**Table S3. Search Strategy of diagnosis analysis and prognosis value analysis.**

| Database (Search date) | | Step | Search Strategy | Number of Results |
| --- | --- | --- | --- | --- |
| Diagnostic Value | Embase (2023/3/25) | #1 | 'breast cancer' | 630,940 |
|  |  | #2 | 'detection effect' | 192 |
|  |  | #3 | 'liquid biopsy' | 14,051 |
|  |  | #4 | 'circulating tumor cell' | 15,301 |
|  |  | #5 | #1 AND #2 AND #3 AND #4 | 1 |
|  | MEDLINE (2023/3/25) | #6 | #5 AND [medline]/lim NOT ([embase]/lim AND [medline]/lim) NOT ([embase classic]/lim AND [medline]/lim) | 0 |
|  | Pubmed (2023/4/27) | 1 | (((circulating tumor cell) AND (liquid biopsy)) AND (detection effect)) AND (breast cancer) | 50 |
| Prognosis Value | Cochrane library (2023/4/27) | #1 | 'CTCs OR circulating tumor cells' | 1,497 |
|  |  | #2 | 'prognosis OR survival' | 153,231 |
|  |  | #3 | 'breast cancer OR breast carcinoma' | 44544 |
|  |  | #4 | ((CTCs OR circulating tumor cells) AND (prognosis OR survival) AND (breast cancer OR breast carcinoma)):ti,ab,kw (Word variations have been searched) | 222 |
|  | Embase (2023/4/27) | #1 | 'ctcs' OR 'circlulating tumors' | 9,955 |
|  |  | #2 | 'prognosis' OR 'survival' | 2,874,694 |
|  |  | #3 | 'breast cancer' OR 'breast carcinoma' | 676056 |
|  |  | #4 | #1 AND #2 AND #3 | 1,521 |
|  |  | #5 | #4 AND 'Article'/it | 625 |
|  | MEDLINE (2023/4/27) | #6 | #5 AND [medline]/lim NOT ([embase]/lim AND [medline]/lim) NOT ([embase classic]/lim AND [medline]/lim) | 47 |
|  | Pubmed (2023/4/27) | 1 | (CTCs OR circulating tumor cells) AND (prognosis OR survival) AND (breast cancer OR breast carcinoma) | 157 |
