# Supplementary material for: Factors influencing the diagnostic and prognostic values of circulating tumor cells in breast cancer: a meta-analysis of 8,935 patients
Source: Front Oncol. 2023 Nov 27;13:1272788. doi: 10.3389/fonc.2023.1272788 (PMC10711619; doi:10.3389/fonc.2023.1272788)
Supplement: Supplementary file 9 [file Table_4.docx]

**Table S4. QUADAS-2 results for Studies Meeting Search Inclusion Criteria**

| Study | Risk of bias | | | | Applicability concerns | | |
| --- | --- | --- | --- | --- | --- | --- | --- |
|  | Patient selection | Index test | Reference standard | Flow and timing | Patients selection | Index test | Reference standard |
|  |  |  |  |  |  |  |  |
| Sabine Riethdorf., 2007 | L | H | L | L | H | L | L |
| Takeshi Sawada., 2016 | H | H | L | L | L | L | L |
| Yuan Sheng., 2017 | H | L | L | L | L | L | L |
| J B Li.,  2017 | H | L | L | L | L | U | L |
| Lidan Jin., 2020 | L | L | L | L | L | U | L |
| Yang L.,  2018 | L | L | L | L | L | L | L |
| Fu-Rong Li., 2013 | L | L | L | L | L | L | U |
| Ulrike Weissenstein., 2012 | L | H | L | L | L | L | U |
| Xiaofen Zhang., 2021 | L | L | L | L | L | L | L |
| Seung Jin Kim., 2011 | L | L | L | L | L | L | L |
| Yan Chen., 2010 | L | L | L | L | L | L | L |
| Shu Zhao., 2013 | L | L | L | L | L | L | L |

**Abbreviation:** L: Low Risk; H: High Risk; U: Unclear Risk
