# Supplementary material for: Factors influencing the diagnostic and prognostic values of circulating tumor cells in breast cancer: a meta-analysis of 8,935 patients
Source: Front Oncol. 2023 Nov 27;13:1272788. doi: 10.3389/fonc.2023.1272788 (PMC10711619; doi:10.3389/fonc.2023.1272788)
Supplement: Supplementary file 10 [file Table_5.docx]

**Table S5. Quality assessment of the included studies’ Bias**

| Study | D1 | D2 | D3 | D4 | D5 | Overall bias |
| --- | --- | --- | --- | --- | --- | --- |
| Riethdorf et al., 2007 | Low | Low | Low | Some concerns | Low | Some concerns |
| Sawada et al., 2016 | High | Low | Low | High | Low | High |
| Sheng et al., 2017 | High | Low | Low | Some concerns | High | High |
| Li et al., 2017 | High | Low | Low | Low | Low | Low |
| Jin et al., 2020 | Low | Low | Low | Low | Low | Low |
| Li et al., 2018 | Low | Low | Low | Low | Low | Low |
| Li et al., 2013 | Low | Low | Low | Low | Low | Low |
| Weissenstein  et al., 2012 | Low | Low | Low | Low | Low | Low |
| Zhang et al., 2021 | Low | Low | Low | Low | Low | High |
| Kim et al., 2011 | Low | Low | Low | Low | Low | Low |
| Chen et al., 2010 | Low | Low | Low | Low | Low | Low |
| Zhao et al., 2013 | Low | Low | Low | Low | Low | Low |

D1: Bias arising from the randomization process; D2: Bias due to deviations from intended interventions;

D3: Bias due to missing outcome data; D4: Bias in measurement of the outcome; D5: Bias in selection of the reported results

Low risk of bias: The study is judged to beat low risk of bias for all domains for this result.

Some concerns: The study is judged to raise some concerns in at least one domain for this result, but not to beat high risk of bias for any domain.

High risk of bias: The study is judged to beat high risk of bias in at least one domain, or to have some concerns for multiple domains in away that substantially lowers confidence in the result.
