# Supplementary material for: Factors influencing the diagnostic and prognostic values of circulating tumor cells in breast cancer: a meta-analysis of 8,935 patients
Source: Front Oncol. 2023 Nov 27;13:1272788. doi: 10.3389/fonc.2023.1272788 (PMC10711619; doi:10.3389/fonc.2023.1272788)
Supplement: Supplementary file 11 [file Table_6.docx]

**Table S6. New Castle-Ottawa Scoring of prognosis analysis for Studies Meeting Search Inclusion Criteria**

| Study | Subject Selection Max 4 | Study Comparability Max 2 | Assessment of Outcomes Max 3 | Total Score |
| --- | --- | --- | --- | --- |
| Radovich et al., 2020 | 4 | 2 | 2 | 8 |
| Cristofanilli et al., 2004 | 4 | 2 | 3 | 9 |
| Bidard et al., 2021 | 4 | 1 | 3 | 8 |
| Moore et al., 2021 | 4 | 2 | 3 | 9 |
| Smerage et al., 2021 | 4 | 1 | 3 | 8 |
| Trapp et al., 2018 | 4 | 1 | 2 | 7 |
| Pang et al., 2021 | 4 | 2 | 3 | 9 |
| Wallwiener et al., 2012 | 4 | 2 | 3 | 9 |
| Hall et al., 2016 | 4 | 1 | 3 | 8 |
| Ramirez et al., 2014 | 3 | 2 | 3 | 8 |
| Jacot et al., 2019 | 4 | 2 | 3 | 9 |
| Pierga et al., 2015 | 4 | 1 | 3 | 8 |
| Jueckstock et al., 2016 | 4 | 1 | 3 | 8 |
| Hayes et al., 2006 | 4 | 1 | 3 | 8 |
| Mu et al., 2015 | 4 | 2 | 3 | 9 |
| Rack et al., 2014 | 4 | 2 | 3 | 9 |
| Larsson et al., 2018 | 4 | 2 | 3 | 9 |
| Yang et al., 2022 | 4 | 2 | 3 | 9 |
| Shiomi-Mouri et al., 2013 | 4 | 1 | 2 | 7 |
| Dawood et al, 2008 | 4 | 1 | 3 | 8 |
| Karhade et al., 2014 | 4 | 2 | 3 | 9 |
| Morales et al., 2018 | 4 | 2 | 2 | 8 |
| Hayashi et al., 2011 | 4 | 2 | 3 | 9 |
