# Supplementary material for: Factors influencing the diagnostic and prognostic values of circulating tumor cells in breast cancer: a meta-analysis of 8,935 patients
Source: Front Oncol. 2023 Nov 27;13:1272788. doi: 10.3389/fonc.2023.1272788 (PMC10711619; doi:10.3389/fonc.2023.1272788)
Supplement: Supplementary file 12 [file Table_7.docx]

| Parameter | Category | Studies | Sensitivity | p1 | Specificity | p2 | LRTChi2 | Pvalue | I^2^ | I^2^lo | I^2^hi |
| --- | --- | --- | --- | --- | --- | --- | --- | --- | --- | --- | --- |
| Country^a^ | Yes | 8 | 0.78  (0.70-0.86) | 0.99 | 0.98  (0.94-1.00) | 0.05 | 3.55 | 0.17 | 44 | 0 | 100 |
|  | No | 4 | 0.63  (0.47-0.79) | - | 0.97  (0.89-1.00) | - | - | - | - | - | - |
| Year^b^ | Yes | 6 | 0.82  (0.75-0.89) | 0.87 | 0.92  (0.80-1.00) | 0.36 | 8.72 | 0.01 | 77 | 50 | 100 |
|  | No | 6 | 0.64  (0.53-0.74) | - | 0.99  (0.98-1.00) | - | - | - | - | - | - |
| Stage^c^ | Yes | 3 | 0.79  (0.64-0.94) | 0.67 | 0.94  (0.79-1.00) | 0.71 | 0.73 | 0.7 | 0 | 0 | 100 |
|  | No | 8 | 0.72  (0.61-0.83) | - | 0.98  (0.93-1.00) | - | - | - | - | - | - |

**Table S7. Results of meta-regression analysis exploring source of heterogeneity for CTC diagnostic efficacy**

**Note:** Based on the logic of programming language for meta-analysis in Stata (Version 12.0), the meta-regression and subgroup analysis could only be divided into two group according to information. In our analysis the subgroup was defined by the follow questions:

^a^ Is it a Chinese research? Yes or No.

b Was it conducted after 2015? Yes or No.

c Was the BC stage advanced stage in the research? Yes or No.

Additionally, one research was excluded due to the cancer stage information missed [1].

Reference:

1. Sheng Y, Wang T, Li H, et al. Comparison of analytic performances of Cellsearch and iFISH approach in detecting circulating tumor cells. Oncotarget. 2017;8(5):8801-8806.
