# Supplementary material for: Factors influencing the diagnostic and prognostic values of circulating tumor cells in breast cancer: a meta-analysis of 8,935 patients
Source: Front Oncol. 2023 Nov 27;13:1272788. doi: 10.3389/fonc.2023.1272788 (PMC10711619; doi:10.3389/fonc.2023.1272788)
Supplement: Supplementary file 13 [file Table_8.docx]

**Table S8. Results of meta-regression analysis exploring source of heterogeneity with OS and PFS/DFS**

| Covariates | OS (Univariate analysis) | | |  | PFS/DFS (Univariate analysis) | | |
| --- | --- | --- | --- | --- | --- | --- | --- |
|  |  |  |  |  |  |  |  |
|  | Coefficient | SE | P |  | Coefficient | SE | P |
| Pulication year | -0.02 | 0.03 | 0.54 |  | -0.02 | 0.01 | 0.16 |
| Sample size | -0.0002 | 0.0002 | 0.54 |  | -0.0001 | 0.0001 | 0.36 |
| Age^a^ | -0.02 | 0.04 | 0.66 |  | -0.02 | 0.02 | 0.18 |
| Continent | -0.26 | 0.19 | 0.18 |  | -0.29 | 0.10 | 0.01 |
| Detection system | -0.77 | 0.30 | 0.01 |  | -0.08 | 0.19 | 0.67 |
| CTC^+^ definition | 0.19 | 0.21 | 0.36 |  | -0.05 | 0.12 | 0.72 |
| Cancer stage | 0.30 | 0.26 | 0.26 |  | 0.09 | 0.14 | 0.54 |
| Therapy | -0.11 | 0.18 | 0.54 |  | -0.10 | 0.09 | 0.30 |
| Sample time | 0.06 | 0.18 | 0.71 |  | -0.01 | 0.09 | 0.93 |
| Follow-up time^b^ | -0.26 | 0.26 | 0.32 |  | -0.12 | 0.14 | 0.42 |

**NOTE**: The dependent variable is the lnHR for PFS/DFS or OS from each study.Weights have been assigned according to the estimated variance of the lnHR. SE, standard error of the coefficient.

^a^ The meta regression analysis based on the other available age information due to the information missing in cohort 4, 5, 11, 14, 16; ^b^ The meta regression analysis based on the other available follow-up time information due to the information missing in cohort 10, 19, 20, 22.
